# Menopausal hormone therapy and exercise on psychosocial well-being and stress in menopausal women

**DOI:** 10.3389/frph.2025.1582406

**Published:** 2025-08-11

**Authors:** Ming Jun Kuck, Eef Hogervorst

**Affiliations:** School of Sports Exercise and Health Sciences, Loughborough University, Loughborough, United Kingdom

**Keywords:** menopausal hormone therapy, physical activity, yoga, mindfulness, psychosocial quality of life, perceived stress

## Abstract

**Introduction:**

Menopausal hormone therapy (MHT) is widely used to alleviate menopausal symptoms, but concerns regarding its risks have led many women to seek alternative treatments, such as physical activity, mindfulness, and yoga. While research suggests that these non-pharmaceutical interventions may improve quality of life (QoL), their efficacy remains debated. This study investigates the independent and interactive effects of MHT and exercise on psychosocial QoL and perceived stress in menopausal women.

**Methods:**

A cross-sectional online survey recruited 272 women aged 40–60 experiencing menopausal symptoms. Participants reported MHT use and engagement in physical activity, mindfulness, or yoga. Psychosocial QoL was assessed using the Menopause-Specific Quality of Life (MenQoL) scale, and perceived stress was measured using PSS-10. Data were analysed using general linear models and partial correlation analyses.

**Results:**

MHT use was significantly associated with poorer psychosocial QoL and worse memory complaints. However, physical activity and yoga were linked to lower perceived stress and better psychosocial QoL. In contrast, mindfulness, as a standalone therapy, was associated with higher perceived stress and depression. No significant interaction effects were observed between MHT and exercise.

**Conclusion:**

While MHT remains a common treatment for menopausal symptoms, its association with poorer psychosocial QoL and memory issues suggests a need for personalised approaches. Engaging in physical activity and yoga appears to offer greater mental health benefits, whereas mindfulness alone is associated with worse mental health. Future research should explore the mechanisms underlying these relationships and the long-term effects of MHT and exercise, especially in early and surgical menopausal women.

## 1 Introduction

Menopausal hormone therapy (MHT) has been shown to relieve symptoms such as hot flushes and night sweats (known together as vasomotor symptoms or VMS) and possible related sleep problems, which the majority of menopausal women experience and struggle with (1). However, controversies surrounding MHT regarding its risk (such as cardiovascular disease or CVD, dementia, breast cancers, etc) highlighted in the publication of the Women's Health Initiative (WHI) study in 2002 have led to a negative perception of the therapy globally, resulting in a significant decline in its use (2–4). Over 60% of women complain of mental health issues, poor cognitive function and lower quality of life in the menopausal transition (1, 5) However, MHT's effect on mental health issues and cognitive complaints is not clear and it is not recommended as a first line treatment to alleviate these issues (5). Furthermore, VMS can impact on CVD, dementia and cognitive impairment risk and may not be adequately treated by non MHT treatments (6–8). These complex challenges have not been adequately addressed via potential other treatments to improve mental health and quality of life (QoL) of menopausal women.

However, extensive research has been conducted to explore alternative treatments to MHT for symptomatic menopausal women with VMS. This body of work has resulted in an increased adoption of non-pharmaceutical interventions, including cognitive behavioural therapy, mindfulness, yoga, and physical activity, aimed at managing menopausal symptoms (9). The efficacy of these interventions remains a topic of debate, largely due to the inconsistencies and lack of high-quality evidence in existing studies. For example, a systematic review and meta-analysis encompassing eight randomised controlled trials (RCT) indicated a positive impact of exercise on both physical and psychological QoL scores in women experiencing menopausal symptoms (10). However, for yoga interventions specifically, although a beneficial effect was observed in the physical QoL domain, these did not yield improvements in psychosocial QoL for menopausal women (10). Similarly, mindfulness-based interventions demonstrated efficacy primarily in alleviating physical and VMS but without significant benefits for psychosocial QoL, as reported in Chen et al.'s (11) meta-analysis. A recent review, Money et al. (12) concluded that there was insufficient evidence to recommend a specific form of exercise for managing menopausal symptoms, particularly regarding vasomotor and psychological symptoms.

To date, limited studies have looked into the interaction between MHT and exercise on cognition, mental health, and hippocampal plasticity in menopausal women (13). Despite the controversies in research, there has been a noticeable rise in the number of menopausal women, specifically those aged 50–59 in the UK, who are looking to start MHT (14). This change in attitude has already led to a shortage in MHT in the UK (15, 16). This trend indicates a growing acceptance of MHT as research continues to evolve. However, there is still much to learn about the potential benefits and risks of MHT, especially when combined with exercise. The updated guidelines from NICE clarified that, while MHT entails certain risks, it treats vasomotor symptoms and is not expected to significantly affect women's overall life expectancy (17). Therefore, this study aims to investigate not only the individual effects of MHT and exercise on the psychosocial QoL (which includes symptoms of depression, anxiety, and memory issues) of menopausal women, but also the interactive effects between MHT and exercise. Previous work also found that women who use MHT tend to be more active and have healthier profiles, which is a major bias in investigating risks and benefits (18).

### 1.1 Aim

This study aims to investigate the independent and interactive effects of exercise and MHT usage on perceived stress, mood and memory complaints, and overall psychosocial QoL (the sum score of mood and memory complaints combined) as dependent variables.

## 2 Methods

### 2.1 Participants and data collection

Middle-aged individuals (40–60 years old) experiencing menopausal symptoms were recruited through convenience sampling. An online survey was accessible through a QR code on social media and menopause-related web groups. No incentives were provided, and all data collected were anonymous after ethical approval was obtained. The study was approved by the Loughborough University Ethics Review Sub-Committee (Project ID: 13264). Participants provided consent without disclosing any identifying information after reviewing an information sheet and were then invited to complete an anonymous online survey. They could withdraw at any time, and their data was then not retained. Demographic data, such as age, ethnicity, and level of education, were collected alongside menopausal stage. The STRAW criteria were applied, and women selected the option that best described their bleeding patterns. Accordingly, the premenopausal was defined as no change in menstrual cycle patterns; early perimenopause as fluctuations in the menstrual cycle in the past year with experience of menstrual bleeding within the last 3 months, late perimenopausal as menstrual cycle fluctuations in the past year with no experience of menstrual bleeding within the last 3 months, and postmenopausal as having had the final menstrual period more than 12 months ago (26).

### 2.2 Menopausal hormone therapy (MHT)

A list of MHTs was provided to participants, who could indicate if they were currently using it (‘2’), had used it in the past (‘1’), or were not using any MHT (‘0’). However, because of the unequal and limited sample size, past MHT users are categorised alongside current MHT users. Analyses showed no differences in this categorisation vs. using individual categories.

The listed MHTs included the following: Oral progesterone, Oral estradiol, Oral Premarin, Estrogen patch, Estrogel, Injection estrogen, or Testosterone. In addition, information on the age at which hormone therapy was initiated and the age at which MHT was discontinued was also gathered, accompanied by the following questions: “What age did you start hormone therapy?” and “If you have stopped hormone therapy, at what age did you stop?” However, insufficient participant numbers did not allow analyses of these potentially modifiable variables.

### 2.3 Type of exercise

Data on the types of exercise practised were gathered through the question: “Do you participate in any of the following mind/body practices? (Yes/No. Please tick all that apply)” The three main grouped options provided included physical activity, mindfulness, yoga, and none. Physical activities included organised sports, exercise classes, running, walking, gym sessions, housework, and other activities. Mindfulness practices included meditation, journaling, breathing exercises, and mindfulness-based practices. Yoga included any yoga classes or yoga exercise engagement.

Women may select more than one type of exercise (e.g., physical activity and yoga, yoga and mindfulness, physical activity and mindfulness, or all three types of exercise). These were were further collapsed into categories for further data analysis. The additional categories were, [apart from ‘None’ (‘0’) or ‘Engaging in 1 type of exercise (‘1’) so either physical activity, yoga or mindfulness’]: ‘Any two of the combined exercises (‘2’)’ and ‘All three exercises (‘3): so women who engaged in physical activity, yoga, and mindfulness’.

### 2.4 Psychosocial quality of life and psychological complaints

Psychosocial quality of life (QoL) was assessed using the Menopause-Specific Quality of Life (MenQoL) scale developed by Hilditch et al. (19). The psychosocial domain of the scale includes 7 items which were used independently and summed up to provide a total Psychosocial QoL. The items were: feeling dissatisfied with personal life, experiencing anxiety or nervousness, having poor memory, accomplishing less than usual, feeling depressed or blue, being impatient with others, and desiring to be alone. Participants indicated whether they had experienced each problem in the past month by selecting ‘Yes’ or ‘No’ and then rated how bothered they were, scoring from “not at all bothered” to “extremely bothered.” The conversion score ranged from 1 to 8. All calculations were based on the official scoring guide. Higher scores on the scale represent a poorer quality of life and a more severe perception of symptoms. The other dependent variables used in analyses, such as memory complaints, anxiety, and depression, were also derived from the psychosocial domain of the MenQoL. The Cronbach's alpha coefficient for the psychosocial domain was *α* = 0.83, indicating good internal consistency.

### 2.5 Perceived stress

The perceived stress scale (PSS-10) by Cohen et al. (20) is a 5-point Likert scale designed to evaluate individuals’ experiences of stress-related feelings and thoughts over the past month. This scale includes four positive and six negative items, allowing women to rate each item on a scale from 0 (never) to 4 (very often). Scores for the four positive items are reversed before calculating the total score, with higher scores reflecting greater perceived stress. The Cronbach's Alpha reliability for this scale was found to be *α* = .87 in the current study.

### 2.6 Data analysis

All data were processed with the IBM Statistical Package for the Social Sciences (SPSS), version 29. Preliminary analyses were conducted to ensure no assumptions were violated for the following tests. A partial correlation analysis was applied to measure the associations between MHT use and the following dependent variables: psychosocial QoL; depression; anxiety; memory issues (all from the MenQoL), and perceived stress, whilst controlling for age and menopausal stage using the STRAW criteria. A general linear model was applied to investigate the interaction between MHT use (current; past, never), activity (none, physical activity, yoga or mindfulness and in separate analyses these individual variables as well as the specific combination of either 2 activities, engaging in any of 2 activities and engaging in all 3 activities), menopausal stage, and age on the dependent variables. For all calculations, *P* < 0.05 was considered statistically significant. Initially, all permutations and combinations of various activities were included for the type of exercises, but these were not significant (possibly due to a lack of power). Ultimately, only the effects of engaging in mindfulness, yoga, physical activity, engaging in any of the two exercises or engaging in all three exercises vs. not participating in any activities were analysed against MHT use (none vs. past and current users). Age and STRAW criteria (menopausal stage) were not significant in these models, nor were interactions of these covariates with MHT and/or engaging in activities. The analysis reflected outcomes without these covariates.

## 3 Results

### 3.1 Participants’ general characteristics and relationship among variables

The Table 1 below provides an overview of the participants’ demographic characteristics. The average age of women included was 51.3 years (SD = 4.8). Nearly half of the women reported being perimenopausal (*N* = 120, 45%), and over half were currently using or had used MHT (60.7%). The majority identified as White British or White other (90.3%), were married or in a relationship (75.5%), were employed full time (55.8%), and had 2–4 children (59.5%). Only 9 (3.3%) of the women had received less than a high school education. No significant differences were observed in the participants’ psychosocial quality of life based on their demographic characteristics.

**Table 1:** Sample characteristics (*n* = 272).

Sample characteristics	*N* (%)
Race/Ethnicity	
White British/Other White Background	246 (90.4%)
Black/ African/Caribbean/ Black British	3 (1.1%)
Asian/Asian British	16 (5.9%)
Mixed/Multiple Ethnic groups	4 (1.5%)
Other	3 (1.1%)
Level of education	
Less than high school	10 (3.7%)
High school/College/A-Levels	86 (31.6%)
University degree	99 (36.4%)
Postgraduate degree	77 (28.3%)
Employment Status	
Employed full-time	153 (56.3%)
Employed part-time	69 (25.4%)
Retired	8 (2.9%)
Other	41 (15.1%)
Marital status	
Married/In a relationship	206 (75.7%)
Divorced/Separated/Single	62 (22.8%)
Widowed	3 (1.1%)
Number of children	
None	62 (22.8%)
1	44 (16.2%)
2–4	161 (59.2%)
More than 4	5 (1.9%)
MHT use	
None	107 (39.3%)
Past	22 (8.1%)
Current	143 (52.6%)
Menopausal stage	
Premenopausal	37 (13.6%)
Perimenopausal	120 (44.1%)
Postmenopausal	115 (42.3%)
Psychosocial symptoms^1^	
Being dissatisfied with my personal life	145 (53.9%)
Feeling anxious or nervous	200 (73.5%)
Experiencing poor memory	202 (74.3%)
Accomplishing less than I used to	179 (65.8%)
Feeling depressed, down or blue	171 (62.9%)
Being impatient with other people	179 (65.8%)
Feelings of wanting to be alone	166 (61.0%)
Exercises	
None	58 (21.3%)
Mindfulness	17 (6.3%)
Yoga	8 (2.9%)
Physical activity	102 (37.5%)
Mindfulness × Yoga	6 (2.2%)
Mindfulness × Physical activity	26 (9.6%)
Yoga × Physical activity	29 (10.7%)
All 3 activities	26 (9.6%)

In our sample, using Spearman's rank correlation, MHT use was associated with poorer Psychosocial QoL (rho = 0.137, *p* = .024) and worse memory issues (with higher scores being more negative for both outcomes, see Table 2). All mental health scores were associated with perceived stress and each other (rho = 0.26- 0.77). Physical activity as a nominal variable was not included in these analyses.

**Table 2:** Spearman's rank correlation between the study's variables.

Variables	1	2	3	4	5	6
MHT use	–					
Perceived stress	.061	–				
Psychosocial QoL	.137^a^	.662^b^	–			
Anxiety	.079	.519^b^	.704^b^	–		
Memory complaints	.155^a^	.257^b^	.629^b^	.342^b^	–	
Depression	.047	.568^b^	.766^b^	.545^b^	.307^b^	–

^a^ Correlation is significant at the 0.05 level (2-tailed).

^b^ Correlation is significant at the 0.01 level (2-tailed).

The analysis using the general linear model (GLM) revealed no significant three-way interaction among exercise type, MHT usage, and either menopausal stage or age in relation to the assessed dependent variables. When menopausal stage and age were excluded as covariates, no significant interaction emerged between MHT usage and exercise type. In the GLM model, exercise type (comparing none to engaging in yoga, physical activity, mindfulness; any 2 or all 3 activities) and MHT usage (comparing non-users to past and current users) were treated as independent variables, as no significant interaction affecting the dependent variables was identified. The individual effects of MHT usage and exercise type on perceived stress, psychosocial quality of life, depression, anxiety, and memory were subsequently explored in greater depth.

**Figure 1:**
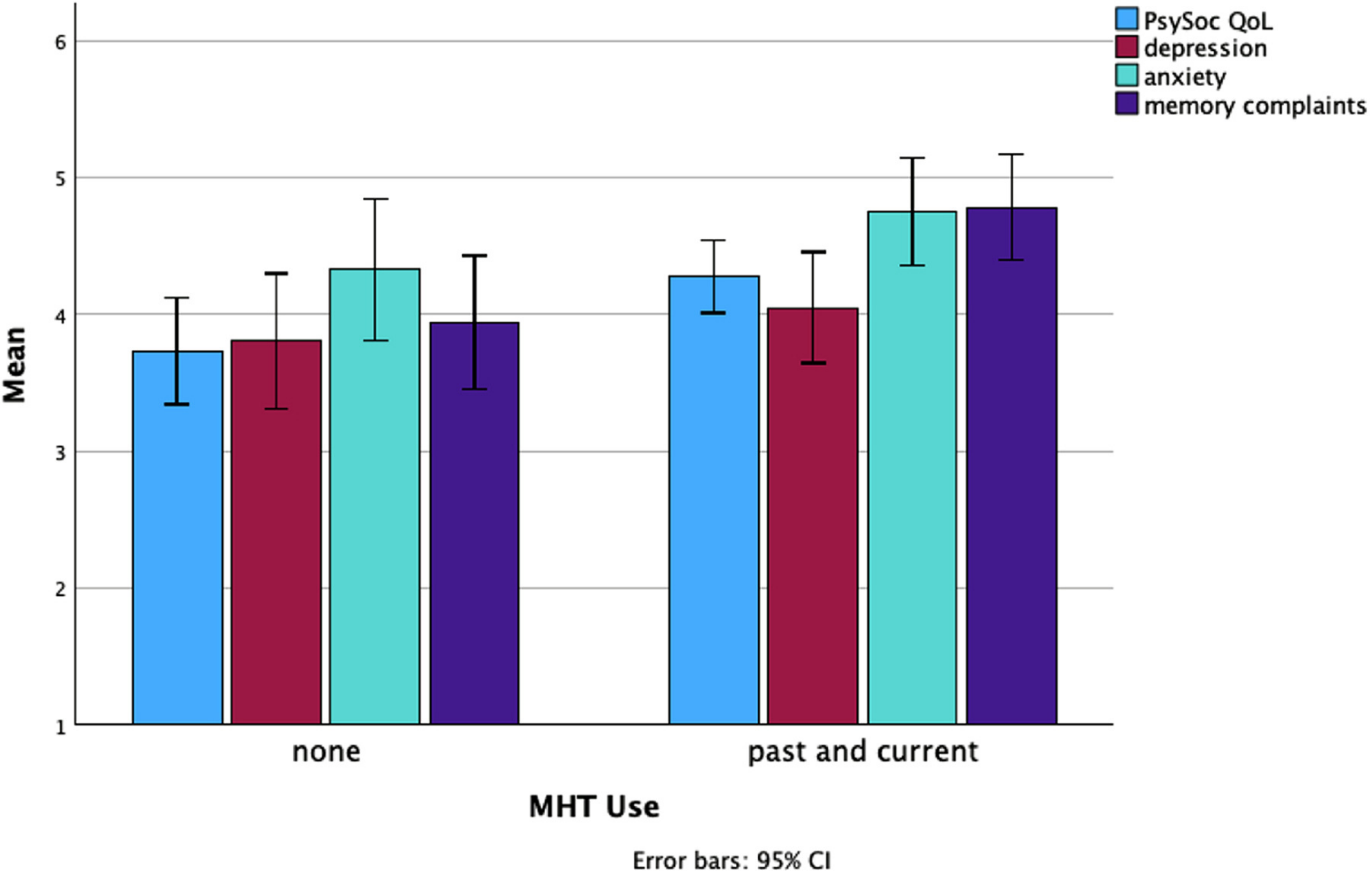
Differences in the levels of psychosocial quality of life, depression, anxiety, and memory complaints between non-MHT users and both past and current users.

In univariate analyses, MHT use was significantly associated with memory complaints, F(1,269) = 6.648, *p* = .010, with past and current users reporting more substantial memory issues (M = 4.78, SD = 2.52) than non-MHT users (M = 3.98, SD = 2.48). Similarly, past and current MHT users (M = 4.27) reported significantly poorer psychosocial QoL compared to non-MHT users (M = 3.74) (see Figure 1). No significant differences were found in the reported levels of depression and anxiety associated with MHT use.

**Figure 2:**
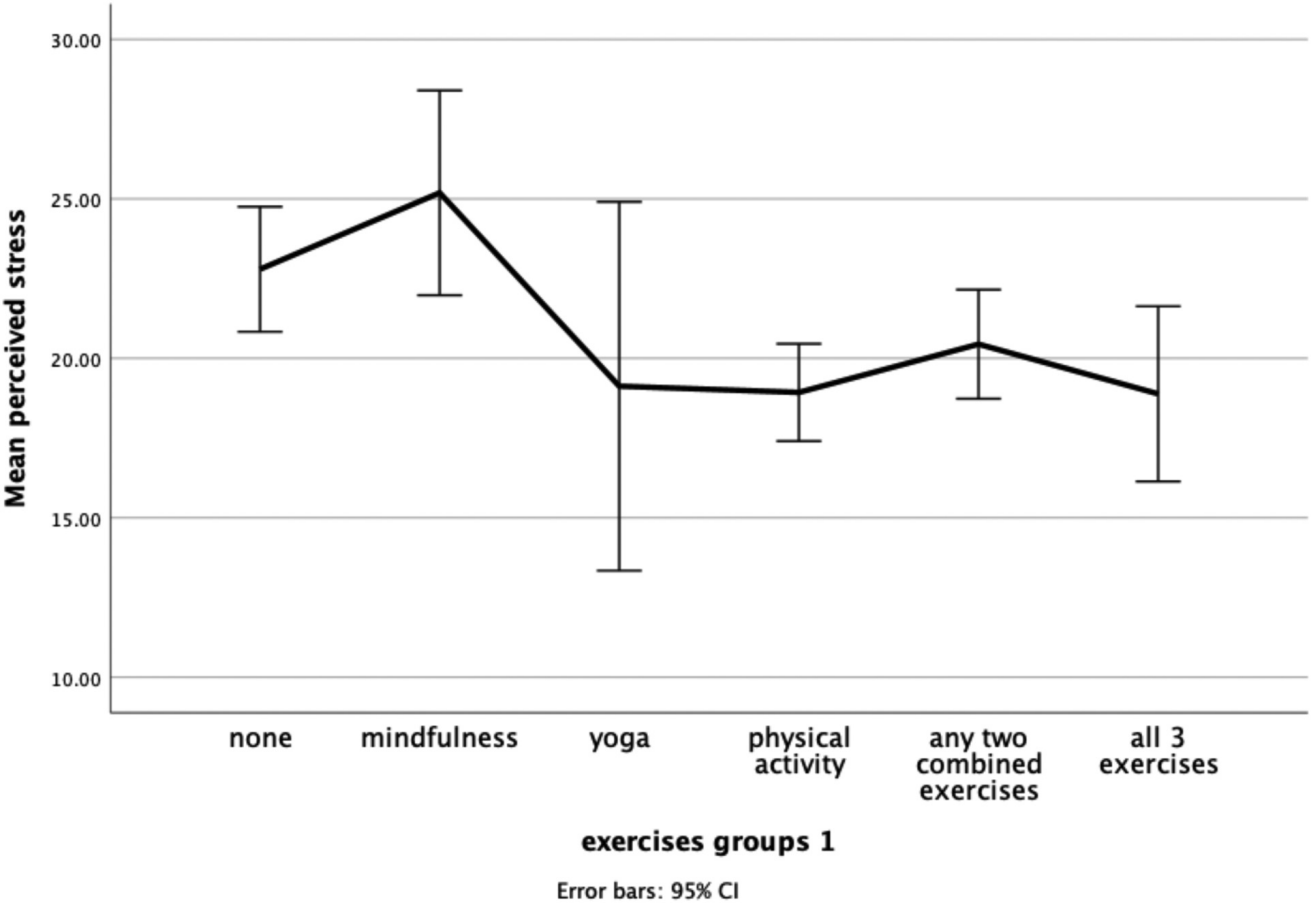
Mean perceived stress levels across different exercise groups.

In univariate analyses, engagement in exercise was significantly linked to women's lower levels of perceived stress and depression. In further *post hoc* analysis using Tukey comparisons, only the relationship between exercise type and perceived stress remained significant: F(7,262) = 3.213, *p* = .003. As shown in Figure 2 below, women who were inactive (M = 22.79, SD = 7.45) or practised mindfulness solely (M = 25.19, SD = 6.02) reported significantly higher levels of perceived stress than women who engaged in physical activity (M = 18.93, SD = 7.73), with a similar trend for yoga.

The type of exercise variable was redefined to better categorize the limited samples in some of the categories. The revised variable, Exercise 1, included the following groups: yoga, mindfulness, physical activity, any two exercises combined (*N* = 61), all 3 exercises combined (*N* = 26), vs. inactive individuals (*N* = 58).

Exercise 1 was significantly associated with psychosocial quality of life (QoL) (*p* = .041), perceived stress (*p* = .003), and depression (*p* = .011). Notably, *post hoc* analyses indicated that women practising mindfulness alone experienced more severe depression symptoms and higher perceived stress levels (see Table 3). While there was an initial significant association between Exercise 1 and psychosocial QoL, subsequent analyses revealed no substantial differences among the individual Exercise 1 groups.

**Table 3:** Mean and standard deviation of mindfulness and physical activity on psychosocial QoL, perceived stress, anxiety, memory complaints, and depression.

Measure	Mindfulness		Physical activity		*p* value
	*M*	*SD*	*M*	*SD*	
Psychosocial QoL	4.88	1.64	3.72	1.85	.051*
Perceived stress	25.19	6.02	18.93	7.73	.040*
Anxiety	6.00	2.09	4.40	2.54	.072
Memory complaints	5.41	2.12	4.19	2.56	.253
Depression	5.44	2.03	3.39	2.61	.027*

* Statistically significant.

Moreover, inactive women showed significantly elevated perceived stress levels (*p* = .033) compared to those who were physically active. They also reported more severe depression symptoms (M = 4.65, SD = 2.62) compared to their physically active counterparts (M = 3.39, SD = 2.61). No significant differences were found between individuals engaging in two or more activities and those who did not.

**Figure 3:**
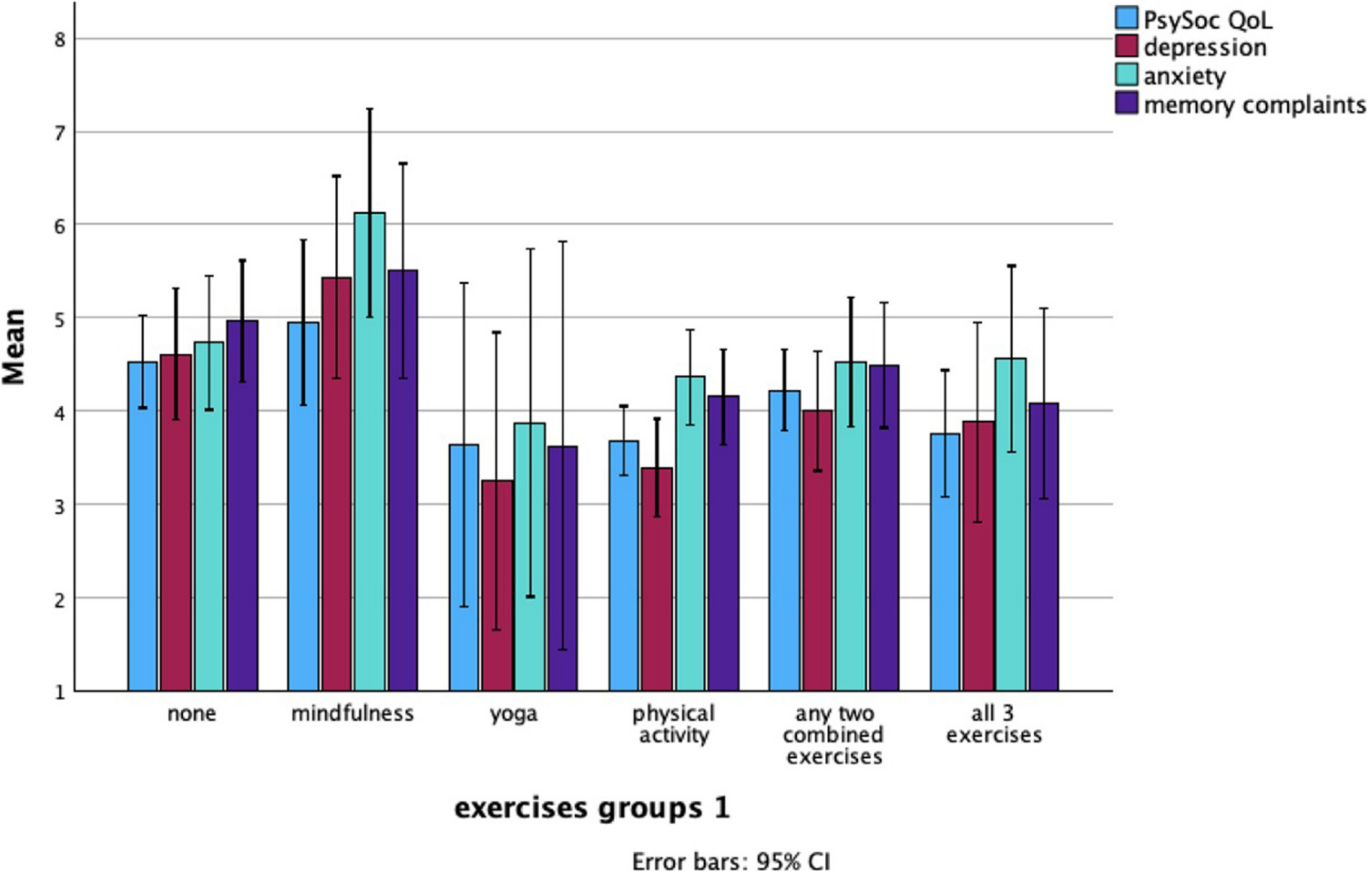
Mean levels of psychosocial QoL, depression, anxiety, and memory complaints across different exercise groups.

Figure 3 illustrates the visual representation of how women who engaged in mindfulness reported the worst average mental health and quality of life, whereas women participating in yoga reported the best quality of life and mental health. Being physically active was better than being inactive in this study.

## 4 Discussion

In contrast to theoretical models and their hypotheses, this cross-sectional study found that MHT users had more memory complaints and worse psychosocial QoL. Additionally, individuals who practised mindfulness or were inactive reported higher levels of perceived stress and depression. However, those who engaged in yoga or physical activity experienced better overall mental health, lower perceived stress, and improved psychosocial QoL.

These findings partially align with broader research on lifestyle interventions. A systematic review and meta-analysis by Liu et al. (21) found that while mindfulness-based interventions (MBIs) were effective in reducing stress, these did not significantly impact anxiety or depression in menopausal women. An alternative explanation for the association observed between higher levels of perceived stress and depression in women who practised mindfulness might be attributed to selection bias. Perhaps these highly stressed women were hopeful that mindfulness could assist them. While MBIs can help alleviate stress in some studies (21), our data suggest these may not offer the same benefits as engaging in physical activity or yoga. Other studies also suggested that these appear to be more effective in managing stress and mood fluctuations during the menopause (22, 23). In contrast with this, a recent review found no significant difference between low- and moderate-intensity exercise—encompassing yoga, walking, exercise training, and other physical activities—in reducing depression and anxiety in menopausal women (24). In our study, anxiety was not affected by any of the independent treatment variables, but depression and stress were positively affected by yoga and engaging in general physical activities. Our data, similar to earlier reviews (1, 5), suggests that MHT does not benefit anxiety, stress and depression.

While findings highlight the potential benefits of lifestyle interventions, many women experiencing mood disturbances and memory complaints may turn to medical treatments such as MHT. Our previous study by Hogervorst et al. (5) found that a 12-month randomised controlled trial (RCT) of MHT (combination estradiol and progesterone) vs. placebo in highly symptomatic menopausal women led to improved well-being, reduced stress, increased activation, and enhanced objective memory. However, these benefits lasted only up to six months before returning to baseline. Conversely, an observational Dutch study reported in the same paper also showed that women using MHT had higher complaints of anxiety and depression. Similarly, in a large registry-based observational study by Vinogradova et al. (25), both women taking MHT and those who would later develop dementia had higher levels of anxiety and depression at baseline. This raises the possibility that MHT may either exacerbate mental health issues or, alternatively, that women with poor mental health and memory complaints are more likely to seek MHT for symptom relief. Thus, the data suggest the potential of self-selection bias, where women experiencing greater psychological distress may be more inclined to use MHT.

The current study has several limitations. The relatively small sample size and self-selection may affect the generalisability of the findings, and the cross-sectional design prevents conclusions about causality. Additionally, the measures regarding exercise type were perhaps too vague to determine the optimal intensity, duration, and specific forms of exercise that could best support mental health. Another limitation is the lack of data on the duration of MHT use and the age at which treatment was initiated, both of which may influence its effects on cognitive and psychological outcomes (5).

In conclusion, this study highlights the complex relationship between MHT, memory complaints, and mental health in menopausal women. While lifestyle interventions like yoga and physical activity show consistent benefits, the effects of MHT remain unclear, with potential reverse causation influencing findings. Future research, particularly long-term RCTs, is essential to determine who may benefit from MHT while minimising potential risks.

## Data Availability

The raw data supporting the conclusions of this article will be made available by the authors, without undue reservation.

## Footnotes

^1^Psychosocial symptoms in Table 3 were measured by the frequency of “yes”.
